# Comprehensive endoscopic management of recurrent esophageal wall abscess revealing concomitant eosinophilic esophagitis

**DOI:** 10.1055/a-2325-2770

**Published:** 2024-06-12

**Authors:** Philippe Onana Ndong, Thierry Piche, Geoffroy Vanbiervliet

**Affiliations:** 1 Service de Gastroentérologie, Hôpital L’Archet 2, Centre Hospitalier Universitaire de Nice, Nice, France

Esophageal wall abscesses (EWAs) are rare lesions often linked to post-traumatic or inflammatory causes. To date, no authors have described their association with a diagnosis of eosinophilic esophagitis (EoE). While typically managed surgically owing to expertise constraints, this approach carries invasive risks, including mediastinitis. Interventional endoscopy offers a less invasive alternative, aiding both comprehensive treatment and etiologic diagnostic evaluation. Here, we report a case of recurrent EWA post-surgical debridement, treated endoscopically via wall incision, purulent content aspiration, and complete abscess fenestration into the esophageal cavity. Surprisingly, endoscopy revealed concomitant EoE.

A 24-year-old man presented with septic shock from a recurrent EWA, 11 cm in length, which had undergone previous surgical management. Endoscopic intervention was pursued this time.

Endoscopic ultrasound revealed a confined submucosal collection. Mucosal bulging and a small orifice, possibly from prior trauma or fistulization, were noted ( Video 1 ). A 3-cm esophageal wall incision allowed access to the abscessed cavity for drainage and irrigation ( Fig. 1 ), followed by closure with clips and insertion of a plastic stent for guided healing.

Endoscopic management of recurrent esophageal wall abscess revealing concomitant eosinophilic esophagitis.Video 1

**Fig. 1:**
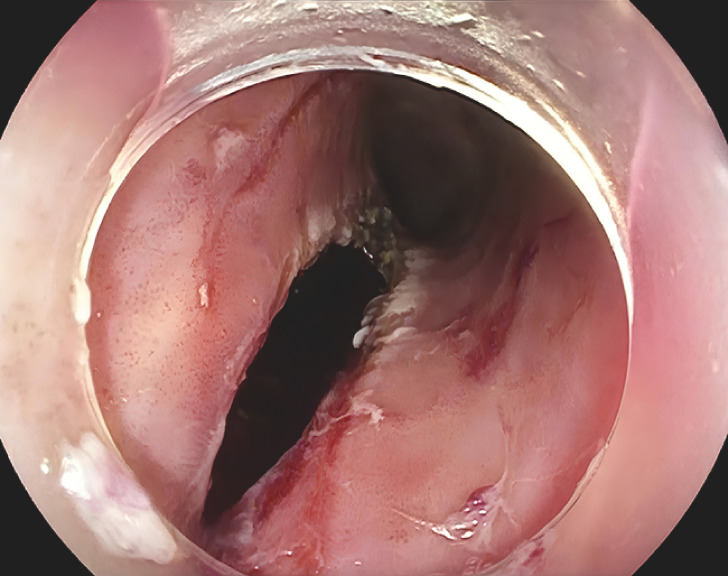
An esophageal incision measuring 3 cm was made to access the abscessed cavity for drainage.

Epithelialization of the treated cavity was observed 3 weeks later. It was separated from the esophageal lumen by a 10-cm long mucosal and fibrous septum ( Fig. 2 ), which was fully sectioned without incident. Endoscopic follow-up at 4 months revealed satisfactory healing, albeit with typical signs of EoE ( Fig. 3 ), with an EREFS (edema, rings, exudate, furrows, stricture) 1 score of 5.

**Fig. 2:**
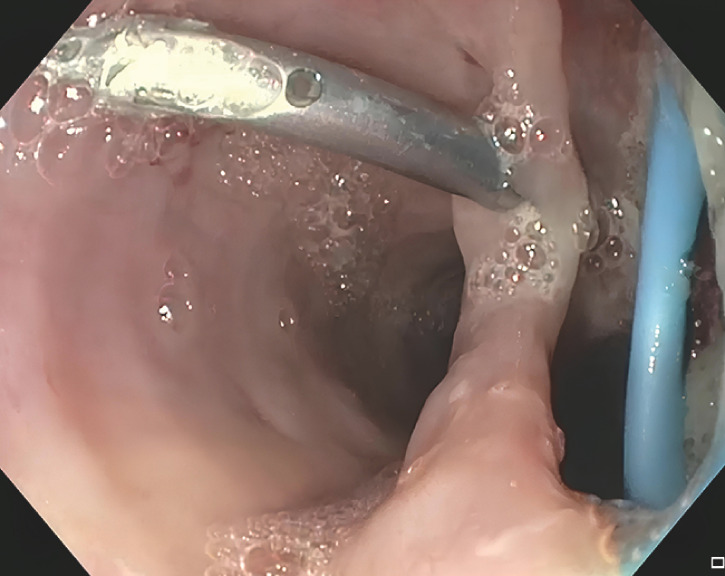
A follow-up at 3 weeks demonstrated a mucosal septum separating the treated cavity from the esophageal lumen.

**Fig. 3:**
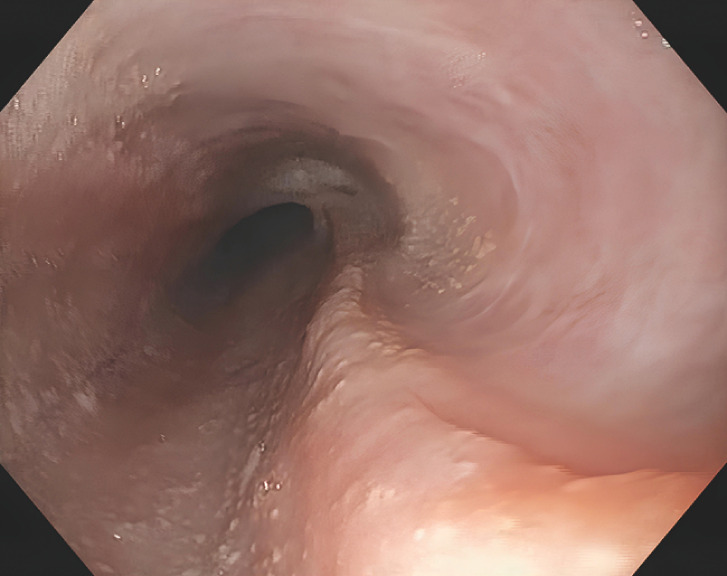
At 4 months, the follow-up revealed healing of the treated section and typical signs of eosinophilic esophagitis.

This case demonstrates that EWAs can be effectively and comprehensively treated in a minimally invasive manner through endoscopy.

Endoscopy_UCTN_Code_CCL_1AB_2AD_3AC

## Notes

Endoscopy E-Videos https://eref.thieme.de/e-videos
*E-Videos* is an open access online section of the journal *Endoscopy* , reporting on interesting cases and new techniques in gastroenterological endoscopy. All papers include a high-quality video and are published with a Creative Commons CC-BY license. Endoscopy E-Videos qualify for HINARI discounts and waivers and eligibility is automatically checked during the submission process. We grant 100% waivers to articles whose corresponding authors are based in Group A countries and 50% waivers to those who are based in Group B countries as classified by Research4Life (see: https://www.research4life.org/access/eligibility/ ). This section has its own submission website at https://mc.manuscriptcentral.com/e-videos .
